# Nationwide Characterization of *MFN2* ‐Related CMT in 176 Japanese Patients: Clinical and Genetic Insights

**DOI:** 10.1002/acn3.70218

**Published:** 2025-09-30

**Authors:** Masahiro Ando, Yujiro Higuchi, Jun‐Hui Yuan, Akiko Yoshimura, Chikashi Yano, Takahiro Hobara, Fumikazu Kojima, Yu Hiramatsu, Satoshi Nozuma, Tomonori Nakamura, Yusuke Sakiyama, Jun Mitsui, Shoji Tsuji, Hiroshi Takashima

**Affiliations:** ^1^ Department of Neurology and Geriatrics Kagoshima University Graduate School of Medical and Dental Sciences Kagoshima Japan; ^2^ Department of Precision Medicine Neurology Graduate School of Medicine, the University of Tokyo Tokyo Japan; ^3^ Department of Neurology The University of Tokyo Hospital Tokyo Japan; ^4^ Institute of Medical Genomics International University of Health and Welfare Chiba Japan

**Keywords:** hmsn (charcot–marie‐tooth), neurogenetics, neuropathy

## Abstract

**Background:**

Mitofusin 2 (*MFN2*) is a major causative gene for axonal Charcot – Marie – Tooth disease type 2A (CMT2A), with a wide phenotypic spectrum. Comprehensive large ‐ scale genotype – phenotype association studies are essential for understanding disease pathogenesis and improved clinical management.

**Methods:**

We conducted a nationwide retrospective study of 176 Japanese patients with genetically confirmed pathogenic or likely pathogenic *MFN2* variants encompassing clinical, electrophysiological, and genetic characterization.

**Results:**

*MFN2* was the second most frequent causative gene among 1211 genetically diagnosed inherited peripheral neuropathy cases in Japan. A total of 76 *MFN2* variants were identified, including nine novel likely pathogenic variants. Disease onset occurred at a mean age of 11.1 years, with distal motor weakness and tibialis anterior involvement as prominent features. Sensory symptoms were present in ~60% of patients and were more common in cases with longer disease duration. Central and systemic features, including pyramidal signs, optic atrophy, and vocal cord paralysis, were also observed. Electrophysiological studies revealed a predominantly axonal sensorimotor pattern, with relatively preserved upper limb conduction and marked lower limb abnormalities. Importantly, 24 patients (16%) were non‐ambulatory, with earlier onset and greater weakness. Domain‐based analysis further revealed that variant location may influence age at onset.

**Conclusion:**

This large‐scale study highlights the genetic and clinical diversity of *MFN2*‐related CMT in Japan. Our findings confirm a motor‐dominant, length‐dependent axonal neuropathy with additional sensory and systemic features in some cases. These results emphasize the importance of early diagnosis, genotype‐informed care, and long‐term follow‐up in managing *MFN2*‐related CMT.

## 1 Introduction

Charcot–Marie–Tooth diseases (CMTs) are the most common inherited peripheral neuropathies (IPNs). Clinically, CMTs are characterized primarily by progressive distal muscle weakness and atrophy, hypoesthesia, areflexia, and foot deformities, and neurologically by deficits in peripheral nerve conduction. However, CMTs are genetically heterogeneous, as advances in next‐generation sequencing have identified over 140 genes associated with various subtypes [1, 2]. This genetic heterogeneity confers substantial variation in clinical course, severity, symptom profile, and even inheritance pattern. Therefore, CMT is traditionally classified based on motor nerve conduction velocity (MNCV) into demyelinating CMT type 1 (MNCV <38 m/s), axonal CMT type 2 (MNCV >38 m/s), and intermediate forms.

Mutation of the gene encoding mitofusion 2 (*MFN2*) is among the most common genetic causes of CMT2, although the relative frequency of *MFN2*‐associated CMT2 (CMT2A2) varies by population [3, 4]. In Japan, it is the most common genetic cause among patients with CMT2 [1, 2, 5]. Mitofusin 2 is a mitochondrial outer membrane GTPase essential for mitochondrial fusion, distribution, and quality control. Thus, dysfunction of MFN2 disrupts mitochondrial dynamics, leading to metabolic impairment. This metabolic disruption is particularly deleterious to long axons innervating lower limb muscles and can ultimately result in axonal degeneration [6].

The clinical phenotype of CMT2A2 is particularly heterogeneous. While many patients present with early‐onset distal muscle weakness and sensory loss, some exhibit additional features such as pyramidal tract signs, optic atrophy, cognitive impairment, or early‐onset stroke [7, 8]. This wide phenotypic spectrum is consistent with a multiplicity of pathogenic mechanisms potentially conferred by genetic heterogeneity and highlights the need for large‐scale genotype–phenotype association studies.

In 2017, we reported a nationwide study analyzing 79 Japanese patients with genetically confirmed *MFN2* variants [5] that confirmed the clinical variability of *MFN2*‐related CMT and revealed several genotype–phenotype associations specific to the Japanese population. However, due to the limited sample size, further investigations are needed to validate and expand upon these findings. In the present study, we analyzed 176 patients with genetically confirmed *MFN2* variants based on genetic testing referrals received from across Japan. This case cohort represents a broad sample of individuals with clinically suspected IPN. We aimed to delineate their clinical features, electrophysiological profiles, and genotypic distribution. Our findings provide valuable information on the natural history and phenotypic spectrum of *MFN2*‐related CMT and may contribute to improving clinical recognition and genetic counseling.

## 2 Materials and Methods

### 2.1 Enrollment Criteria

This study included Japanese patients receiving clinical and genetic evaluation as part of an ongoing nationwide genetic investigation into IPNs/CMT. For patients presenting with demyelinating features, the presence or absence of peripheral myelin protein 22 (*PMP22*) gene duplication or deletion was assessed using either fluorescence in situ hybridization or multiplex ligation‐dependent probe amplification. Patients with confirmed *PMP22*‐related CMT1A or hereditary neuropathy with liability to pressure palsies (HNPP) according to these tests were excluded from the current study cohort. Because *PMP22* testing is performed locally as a routine first‐line diagnostic step covered by national health insurance, the exact number of excluded patients was not available. The study protocol was approved by the Institutional Review Board of Kagoshima University, and written informed consent was obtained from all patients or their legal guardians.

### 2.2 Genetic Analysis

Between 2007 and 2024, we conducted genetic screening on 3314 unrelated Japanese patients clinically diagnosed with IPNs, including CMT, using in‐house panel‐based sequencing. These panels targeted known IPNs/CMT‐related genes, including *MFN2*, and were conducted using one of the following platforms: DNA microarrays (Affymetrix, Santa Clara, CA, USA), Illumina MiSeq (Illumina, San Diego, CA, USA), or Ion Proton (Thermo Fisher Scientific, Waltham, MA, USA). Over the course of panel updates, *MFN2* remained a core gene of interest. Among the 3314 patients, 756 individuals who tested negative for pathogenic variants in initial panel testing subsequently received whole‐exome sequencing. In addition, repeat expansion disorders involving *NOTCH2NLC*, *RFC1*, and *LRP12* were screened in undiagnosed cases using protocols described in our previous publications [9, 10, 11].

Previously reported pathogenic or likely pathogenic variants were identified by referring to the Human Gene Mutation Database Professional. All candidate pathogenic variants were confirmed by Sanger sequencing and interpreted according to the guidelines issued by the American College of Medical Genetics and Genomics (ACMG) [12]. Novel variants were confirmed by cross‐referencing with global population databases such as the Genome Aggregation Database (gnomAD), the Japanese multi‐omics reference panel (jMorp), and our institutional control dataset. In silico predictions of variant pathogenicity were obtained using REVEL, CADD, and BayesDel. In cases where these tools yielded conflicting results, only the REVEL score was used as computational evidence for variant classification (PP3). Although the ClinGen Sequence Variant Interpretation Working Group recommends the use of REVEL as strong‐level evidence when specific thresholds are met, we applied it as moderate‐level support in this study [13]. For segregation analysis (PP1), we adopted the point‐based approach proposed by the ClinGen Sequence Variant Interpretation Working Group, which provides a quantitative framework for scoring segregation evidence in autosomal dominant disorders [14].

### 2.3 Clinical and Electrophysiological Data Collection

This study included 176 patients carrying previously reported or novel *MFN2* variants classified as pathogenic or likely pathogenic according to ACMG guidelines. Clinicodemographic and electrophysiological findings were obtained from medical records submitted by referring institutions. Clinicodemographic data collected included age at onset, family history, patterns of muscle weakness and sensory disturbances, presence and nature of foot deformity, status of tendon reflexes, and ambulatory status. Electrophysiological assessments comprised nerve conduction velocities and compound motor action potential (CMAP)/sensory nerve action potential (SNAP) amplitudes of representative motor and sensory nerves.

### 2.4 Statistical Analyses on Clinical and Electrophysiological Findings

Statistical analyses were performed using GraphPad Prism (version 10.5.0; GraphPad Software, San Diego, CA). Comparisons between two groups were conducted using the Mann–Whitney U‐test, and comparisons among more than two groups were performed using the Kruskal–Wallis test. Associations between continuous variables were assessed using Spearman's rank correlation coefficient. A *p*‐value < 0.05 was considered statistically significant for all tests.

## 3 Results

### 3.1 Genetic Findings

**FIGURE 1:**
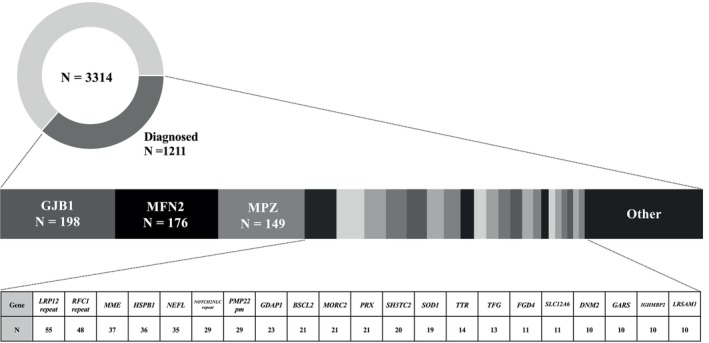
Distribution of genetically diagnosed cases among 3314 Japanese patients. A genetic diagnosis was established in 1211 patients (36.5%). Among these, *MFN2* was identified as the second most common causative gene, accounting for 176 cases (14.5%), following GJB1 (198 cases). Patients with PMP22 duplication or deletion (CMT1A or HNPP) were excluded prior to referral and are therefore not included in this cohort. pm: Point mutation.

Among the 3314 Japanese patients with clinically suspected IPNs/CMT receiving genetic testing, 1211 (36.5%) obtained a definitive molecular diagnosis. Among these genetically diagnosed cases, *GJB1* (encoding gap junction beta‐1 protein, connexin 32) was the most frequently implicated in IPNs/CMT (198 cases), followed by *MFN2* (176 cases). The distribution of all causative genes identified in this Japanese IPNs/CMT cohort is shown in Figure 1. In total, 76 distinct *MFN2* variants were identified, of which nine were novel (p.Ile88Asn, p.His128Gln, p.Thr129Asn, p.Cys217Gly, p.Ser231Tyr, p.Glu308Gly, p.Thr356Pro, p.Glu744Gly, and p.Glu744Val) and classified as likely pathogenic based on ACMG guidelines. Notably, two patients harbored digenic variants involving *MFN2* and another gene, one with a previously reported *PMP22* missense variant and the other with a known missense variant in *SOD1*. These two cases have been described in our prior publications [5, 15]. Details of these nine novel, likely pathogenic variants are summarized in Table 1, and representative pedigrees are shown in Figure S1. Additionally, we identified seven variants of uncertain significance (VUS), which are listed in Table S1. These variants were excluded from the main genotype–phenotype association analyses but warrant further investigation as additional functional or segregation data become available.

**TABLE 1:** Summary of novel *MFN2* variants identified in this study.

Variant number	1	2	3	4	5	6	7	8	9
Nucleotide change	c.263 T>A	c.384C>A	c.386C>A	c.649 T>G	c.692C>A	c.923A>G	c.1066A>C	c.2231A>G	c.2231A>T
Amino acid change	p.Ile88Asn	p.His128Gln	p.Thr129Asn	p.Cys217Gly	p.Ser231Tyr	p.Glu308Gly	p.Thr356Pro	p.Glu744Gly	p.Glu744Val
Number of patients	1	1	1	1	1	1	1	1	1
**Control database**									
gnomAD	—	—	—	—	—	—	—	—	—
jMorp	—	—	—	—	—	—	—	—	—
ACMG_PM2	support	support	support	support	support	support	support	support	support
Our database	—	—	—	—	—	—	—	—	—
ACMG_PS4	support	support	support	support	support	support	support	support	support
**in silico**									
REVEL	0.935	0.811	0.857	0.943	0.935	0.958	0.937	0.942	0.926
CADD	28.4	24	30	27.9	32	31	28.3	28.4	27.5
BayesDel	0.455901	0.462834	0.155956	0.544668	0.5338	0.581864	0.581149	0.513196	0.419994
ACMG_PP 3	moderate	moderate	moderate	moderate	moderate	moderate	moderate	moderate	moderate
**Segregation study**									
Segregation		de novo		de novo	*	de novo		**	**
ACMG_PM 6, PP1, 4				support	support	support		moderate	moderate
**Same residue known as pathogenic**									
Same residue	I88F/V	H128R/Y	T129A/P	C217F/W	S231C/F	E308X	T356A/N/S	E744D/K	E744D/K
ACMG_PM 5	moderate	moderate	moderate	moderate	moderate	moderate	moderate	moderate	moderate
ACMG criteria	Likely pathogenic	Likely pathogenic	Likely pathogenic	Likely pathogenic	Likely pathogenic	Likely pathogenic	Likely pathogenic	Likely pathogenic	Likely pathogenic

*Note:* REVEL scores ≥ 0.773 were considered moderate evidence of pathogenicity, and scores between 0.644 and 0.773 were considered supporting, based on thresholds established by the ClinGen Sequence Variant Interpretation Working Group. REVEL scores below 0.644 were interpreted as not supportive. CADD scores ≥ 25.3 and BayesDel scores ≥ 0.13 were considered indicative of damaging effects. *; Denotes variants that were observed in one affected individual only. ** Denotes variants that were observed in one affected and not observed in one unaffected individual from the same family.

**FIGURE 2:**
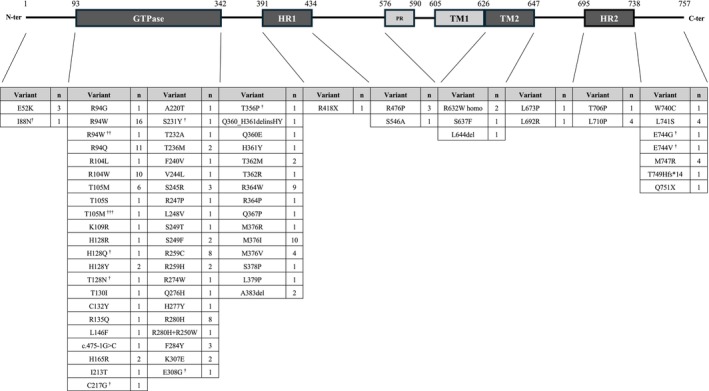
Distribution of *MFN2* variants identified in 176 Japanese patients with Charcot–Marie–Tooth disease. The schematic diagram of the *MFN2* protein shows the location of 76 distinct pathogenic or likely pathogenic variants across functional domains. n: Number of patients carrying the variant, ^†^: Novel variant, ^††^: Digenic mutation with *SOD1* p.Gly38Arg, ^†††^: Digenic mutation with *PMP22* p.Arg159Cys. HR1: Heptad repeat 1 domain, HR2: Heptad repeat 2 domain, PR: Proline‐rich domain, TM1/TM2: Transmembrane domain 1/2.

The identified variants were distributed throughout the entire protein, with a notable concentration within the GTPase domain (106 cases). Variants were also detected in other functional regions, including the heptad repeat domain 1 (HR1; 1 case), HR2 (5 cases), and the second transmembrane domain (TM2; 4 cases). The most frequently observed variants were p.R94W (16 cases) and p.R94Q (11 cases), followed by p.R104W (10 cases) and p.M376I (10 cases). The distribution and frequency of all identified variants categorized by protein domain are shown in Figure 2 and Table 2.

**TABLE 2:** Associations of age at onset with *MFN2* mutation location (codon and predicted protein domain).

Codon	Domain	Onset age	N
1–93		9.8 ± 3.3	4
93–342	GTPase	10.3 ± 11.5	106
342–391		10.7 ± 13.3	37
391–434	HR1	2	1
434–576		30.5 ± 25	4
626–647	TM2	36.3 ± 24.0	4
647–695		8.5 ± 3.5	2
695–738	HR2	4 ± 3.7	5
738–757		8.8 ± 8.4	13

*Note:* Patients were grouped based on the codon location of *MFN2* variants and associated functional domains. Dashes indicate regions outside of annotated domains. HR1 and HR2, heptad repeat domains; TM2, second transmembrane domain; GTPase, GTPase domain. *N* = number of patients with variants in the indicated codon range. Groups were compared using the Kruskal–Wallis test (*p* = 0.099).

### 3.2 Clinical Findings

The mean age at onset among the 176 patients with *MFN2*‐related IPNs/CMT was 11.1 ± 13.0 years. The distribution of onset age across the cohort is illustrated in Figure 33A. The sex distribution was nearly equal, with 87 males and 89 females. A positive family history was identified in 86 cases, while the remaining 87 cases had no reported family history, and information was unavailable for three cases, indicating an approximately equal distribution between familial and apparently sporadic cases.

**FIGURE 3:**
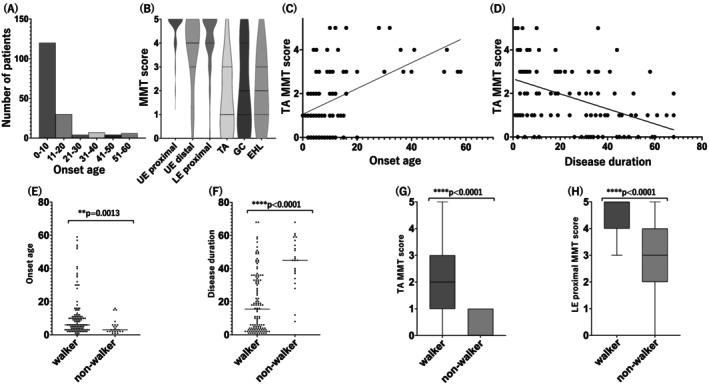
Clinical characteristics and functional associations in *MFN2*‐related CMT. (A) Distribution of age at onset in the cohort. (B) Violin plots showing manual muscle test (MMT) scores for six muscle groups: Upper extremity (UE) proximal and distal, lower extremity (LE) proximal, tibialis anterior (TA), gastrocnemius (GC), and extensor hallucis longus (EHL). Spearman's rank correlation revealed a significant positive correlation in (C) (ρ = 0.485, *p* < 0.0001, *n* = 105) and a significant negative correlation in (D) (ρ = −0.447, *p* < 0.0001, *n* = 104). (E) Onset age was significantly lower in non‐ambulatory (un‐walkable) patients than in ambulatory (walkable) patients. (F) Disease duration was significantly longer in the un‐walkable group. (G, H) TA and lower extremity proximal MMT scores were significantly reduced in the un‐walkable group compared to the walkable group.

Among subgroups with available data, 99.4% (160/161) exhibited distal muscle weakness and 95.9% (142/148) demonstrated distal muscle atrophy. The denominators differ across items because of missing data, as not all clinical parameters were available for every patient. Hypoesthesia was documented in 60.2% of the *MFN2*‐related CMT subgroup with available data (77/128). In addition, deep tendon reflexes were reduced or absent in 92.1% (128/140). Collectively, these findings suggest a motor‐dominant neuropathy. Notably, the mean disease duration in patients with hypoesthesia was 29.2 ± 20.1 years, compared to 18.4 ± 17.0 years for those without sensory involvement, indicating that sensory nerve degeneration progresses more slowly than motor axon damage. In addition to peripheral nerve symptoms, several central or systemic complications were observed in a minority of patients, including pyramidal tract signs in 12 patients, optic atrophy in 5, cataracts in 2, vocal cord paralysis in 4, respiratory failure in 4, and mental retardation or cognitive impairment in 9 patients. Cataracts were observed in only two cases, both harboring the R104W variant, and mental retardation was also noted in two patients with the same variant. Among four patients with the L710P variant, two exhibited pyramidal tract signs. Particularly severe phenotypes were associated with the R364W variant, which was identified in nine patients with very early onset (mean age at onset 2.6 years, range 0–6). In addition to neuropathy, these patients frequently showed other complications, such as optic atrophy (2/9), vocal cord paralysis (2/9), and respiratory muscle involvement (1/9). Electrophysiological findings also indicated severe impairment in patients with the R364W variant, with median motor responses absent in 7/10, tibial motor responses absent in 6/7, median sensory responses absent in 6/7, and sural responses absent in 5/6.

Manual muscle testing (MMT) results of upper extremity (UE) proximal muscles were available for 92 patients, UE distal muscle test results for 93 patients, lower extremity (LE) proximal muscle results for 93 patients, tibialis anterior (TA) test results for 105, gastrocnemius (GC) results for 91, and extensor hallucis longus (EHL) test results for 33 patients. Comparisons revealed that UE proximal muscles were the least severely affected, with a mean MMT score of 4.78 ± 0.53, and deficits were also mild for LE proximal muscles (4.22 ± 1.09) and mild to moderate for UE distal muscles (3.79 ± 1.39). In contrast, among individual lower limb muscles, TA showed the most prominent weakness (1.76 ± 1.50), while GC (2.16 ± 1.81) and EHL (2.06 ± 1.54) were relatively less affected. This distribution of muscle strength scores is shown in Figure 3B. Notably, TA weakness was more pronounced in patients with earlier onset age and longer disease duration (Figure 3C,D). Spearman's rank correlation revealed a significant positive correlation between TA MMT score and onset age (ρ = 0.485, 95% CI: 0.318–0.623, *p* < 0.0001, *n* = 105) and a significant negative correlation with disease duration (ρ = −0.447, 95% CI: −0.593 to −0.273, *p* < 0.0001, *n* = 104), indicating that earlier onset and longer disease duration were associated with more severe TA involvement. Among the 105 patients with available TA MMT data, 70 patients (66.7%) showed severe weakness with MMT scores of 0–2, which we considered to be consistent with clinical foot drop. Among patients with sensory disturbances, touch sensation was impaired in 60% (27/45), pinprick or cold perception in 72.5% (29/40), and vibration sense in 87.9% (51/58). Patients with sensory symptoms had a significantly longer disease duration (29.2 ± 20.1 years, *n* = 77) than those without sensory symptoms (18.4 ± 17.0 years, *n* = 51; *p* = 0.0026, Mann–Whitney U test).

We also assessed the age at initiation of assistive interventions. Among patients with available data, the mean age was 15.2 ± 14.2 years for orthotic use (*n* = 18), 11.1 ± 4.4 years for foot surgery (*n* = 26), and 27.4 ± 17.8 years for wheelchair use (*n* = 18). In these 18 patients, the interval from disease onset to wheelchair dependence ranged from 0 to 54 year. Notably, 4 patients (22.2%) became wheelchair dependent within 10 years after onset, while 8 patients (44.4%) required wheelchairs only after more than 20 years, highlighting marked variability in disease progression. Ambulatory status was assessable in 147 patients, of which 123 (83.7%) were ambulatory and 24 (16.3%) non‐ambulatory at the time of evaluation. Patients who were non‐ambulatory exhibited significantly lower TA and lower limb proximal muscle strength scores, as well as younger onset age and longer disease duration compared to ambulatory individuals (Figure 3E–H).

### 3.3 Electrophysiological Findings

Nerve conduction studies were performed in a subset of patients, and findings were consistent with an axonal sensorimotor neuropathy characterized by predominant lower limb and motor nerve involvement. In the upper limbs, motor conduction parameters were relatively preserved, as indicated by a median nerve MNCV of 49.9 ± 8.2 m/s and CMAP amplitude of 6.4 ± 5.2 mV. Similarly, the ulnar nerve exhibited near‐normal values of 52.7 ± 9.3 m/s and 5.1 ± 4.2 mV, respectively. In contrast, lower limb motor nerves showed profound impairment. For instance, the tibial nerve exhibited a markedly reduced CMAP amplitude of 1.2 ± 2.9 mV despite a moderately preserved MNCV of 37.8 ± 7.2 m/s, suggesting axonal degeneration. Sensory nerve studies revealed mild to moderate involvement. The mean sensory conduction velocity (SCV) of the median nerve was 47.8 ± 8.2 m/s, and SNAP amplitude was 9.5 ± 9.1 μV, suggesting mild impairment. Similarly, mean ulnar nerve SCV was 47.2 ± 8.5 m/s, and SNAP was 9.0 ± 12.4 μV. In contrast, the sural nerve showed greater impairment, as indicated by an SCV of 41.2 ± 7.2 m/s and SNAP of 3.1 ± 5.2 μV. Notably, in several advanced cases, both CMAPs and SNAPs were absent in multiple nerves, highlighting that motor and sensory axons can be severely affected and that strict dichotomization into motor‐dominant or sensory‐predominant forms is not always feasible.

**FIGURE 4:**
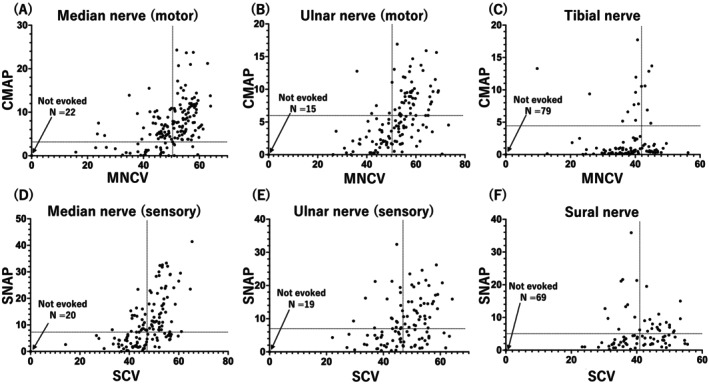
Motor and sensory nerve conduction studies in patients with *MFN2*‐related CMT. (A–C) Scatter plots of compound muscle action potential (CMAP, mV) versus motor nerve conduction velocity (MNCV, m/s) for the median (A), ulnar (B), and tibial (C) nerves. (D–F) Scatter plots of sensory nerve action potential amplitude (SNAP, μV) versus sensory conduction velocity (SCV, m/s) for the median (D), ulnar (E), and sural (F) nerves. Dashed lines represent the approximate lower limits of the normal range for each parameter (Median CMAP > 3.1 mV; Median MNCV > 49.6 m/s; Median SNAP > 7.0 μV; Median SCV > 47.2 m/s; Ulnar CMAP > 6.0 mV; Ulnar MNCV > 50.1 m/s; Ulnar SNAP > 6.9 μV; Ulnar SCV > 46.9 m/s; Tibial CMAP > 4.4 mV; Tibial MNCV > 41.7 m/s; Sural SNAP > 5.0 μV; Sural SCV > 40.8 m/s). Each dot represents a single nerve measurement; some patients contributed data from both sides.

These findings collectively suggest that *MFN2*‐related CMT presents as a motor‐dominant axonal neuropathy in Japanese patients, with relatively spared upper limb nerve function and more severe impairment of lower limb motor nerve conduction (Figure 4A–F).

## 4 Discussion

In this study, we present a comprehensive clinical and genetic analysis of 176 Japanese patients harboring pathogenic or likely pathogenic *MFN2* variants. To our knowledge, this is one of the largest single‐country cohorts of *MFN2*‐related CMT patients reported to date [16, 17, 18, 19]. Among the 1211 genetically diagnosed IPN/CMT cases in our cohort, *MFN2* was the second most frequently implicated gene after GJB1, accounting for 14.5% of cases. Among the 176 cases of confirmed *MFN2*‐associated CMT disease, we identified 76 distinct MFN2 variants, including nine novel missense variants classified as likely pathogenic according to ACMG guidelines. The relatively high frequency of *MFN2* in our Japanese cohort is consistent with prior studies, although the precise prevalence ranking has varied by population [3, 4]. Notably, two patients exhibited digenic inheritance involving *MFN2* and either *PMP22* or *SOD1*, highlighting the genetic complexity underlying inherited neuropathies. These findings reinforce the need for comprehensive genetic screening and careful variant interpretation.

Clinically, the disease manifested at an early age, with a mean age at onset of 11.1 years. While early childhood onset is well documented in *MFN2*‐related CMT [20, 21], our domain‐based analysis revealed a trend toward later onset in patients with variants located downstream of the HR1 domain and within the TM2 region (Table 2). Although this association did not reach statistical significance (Kruskal–Wallis, *p* = 0.099), it nonetheless suggests a modulatory role of variant location on disease severity and progression.

Muscle strength assessments and comparative nerve conduction studies confirmed a predominantly motor‐dominant phenotype with marked weakness of distal lower limb muscles, particularly of the TA, but relative preservation of upper limb strength. This pattern reflects the classic length‐dependent axonal degeneration observed in CMT2A. Greater weakness in the TA was significantly associated with earlier disease onset and longer disease duration, reflecting the cumulative burden of axonal dysfunction over time. This motor‐dominant phenotype may, at least in part, be explained by the selective vulnerability of motor neurons to mitochondrial dysfunction, as selective mitochondrial depletion in motor neurons has been demonstrated despite the ubiquitous expression of MFN2 [22]. Although primarily motor‐dominant, sensory symptoms were observed in approximately 60% of these *MFN2*‐related CMT patients. Among cases with sensory nerve involvement, vibration sense was the most frequently affected modality, followed by pinprick/cold and light touch. These findings suggest a length‐dependent sensory fiber degeneration pattern disproportionately affecting large fibers. Our previous study involving 79 Japanese patients with *MFN2*‐related CMT also reported frequent hypoesthesia, particularly in vibration [5]. While disease duration was not assessed in that study, the present findings confirm and expand upon our previous observations by demonstrating more prevalent sensory involvement in patients with longer disease duration, suggesting slower progression of sensory fiber degeneration compared to motor axons. The underlying mechanisms for this difference warrant further study.

Several patients exhibited other neurological or systemic features in addition to peripheral neuropathy, including pyramidal tract signs, optic atrophy, cognitive impairment, vocal cord paralysis, and respiratory dysfunction. Though less common, these manifestations underscore the potential for *MFN2* mutations to deleteriously affect central or multisystem pathways. These observations are consistent with previous reports describing central nervous system involvement and multisystemic features in *MFN2*‐related disorders, including pyramidal signs, cognitive impairment, optic atrophy, and even cataracts [7, 8, 23]. Recognition of these atypical features is essential for holistic patient evaluation and may prompt further investigations in early‐onset or rapidly progressive cases.

In this cohort, 16% of patients were non‐ambulatory at the time of evaluation. These non‐ambulatory patients exhibited earlier onset, longer disease duration, and more severe lower limb weakness, particularly of the TA and proximal muscles, suggesting that earlier onset is predictive of a more severe clinical course. Additionally, the timing of orthotic support, foot surgery, and wheelchair use suggests that functional decline occurs progressively during childhood and adolescence. However, the proportion of non‐ambulatory patients was lower than reported in a previous study, where 24.5% of *MFN2*‐CMT patients were wheelchair‐dependent [19], possibly reflecting differences in cohort composition, follow‐up duration, or variant spectrum. These findings highlight the importance of early identification and proactive multidisciplinary care.

Consistent with the symptom profile, electrophysiological findings demonstrated a sensorimotor axonal pattern with relatively preserved conduction in the upper limbs and more marked abnormalities in the lower limbs. In particular, median and ulnar nerves frequently showed near‐normal conduction velocities and compound action potential amplitudes, potentially complicating diagnosis if lower limb studies are omitted. Alternatively, tibial and sural nerves were moderately affected, underscoring the diagnostic value of tibial and sural nerve assessments in suspected *MFN2*‐related CMT cases. While the overall deficit pattern was suggestive of sporadic axonal degeneration, a subset of patients exhibited slower conduction velocities suggestive of demyelinating features or combined axonal loss and demyelination. This electrophysiological heterogeneity reflects the broad phenotypic spectrum of *MFN2*‐related neuropathy and the importance of thorough nerve conduction studies.

This study has several limitations. First, the clinical and electrophysiological data were retrospectively collected from multiple institutions, introducing variability in assessment methods and data quality. Also, not all parameters were available for every patient, resulting in relatively smaller samples for evaluation of parameters such as MMT scores, sensory assessments, and functional status. Second, the cross‐sectional nature of the study precluded longitudinal assessment of disease progression. Third, although stringent ACMG criteria were used for variant classification, the pathogenicity of the novel variants remains to be experimentally validated. Fourth, our cohort was established from nationwide genetic testing referrals of patients clinically diagnosed with IPN/CMT. Consequently, patients with other *MFN2*‐related phenotypes, such as isolated distal myopathy [24], amyotrophic lateral sclerosis/frontotemporal dementia (ALS/FTD) [25, 26], or lipomatosis [27], were not included. Therefore, the present findings should be interpreted within the context of CMT/IPN, and may not capture the broader phenotypic spectrum of *MFN2*‐related disorders. Fifth, because genetic testing was not performed for all family members, the penetrance of MFN2 variants in familial cases could not be reliably estimated.

This large‐scale nationwide study of 176 Japanese patients with *MFN2*‐related CMT highlights the importance of *MFN2* for maintaining the survival and conduction of large peripheral axons, particularly those innervating LE muscles. We confirmed a motor‐dominant phenotype with preferential lower limb involvement and showed that earlier onset and longer disease duration are associated with more severe motor impairment and loss of ambulation. Sensory deficits and multisystem involvement were less common, but also tended to be more prevalent in patients with longer disease duration, suggesting that sensory axon degeneration occurs more slowly than motor degeneration. Collectively, these findings reinforce the importance of early diagnosis, comprehensive clinical evaluation, and long‐term follow‐up for the management of *MFN2*‐related CMT. Although no disease‐modifying therapy is currently available, early genetic diagnosis provides valuable prognostic information and will enable timely inclusion of affected individuals in future clinical trials and therapeutic interventions as they emerge. The novel variants identified in this study remain to be experimentally validated.

## Author Contributions

M A, Y H, and H T conceived and designed the study. M A, Y H, J Y, Y C, T H, F K, Y H, S N, T N, and Y S contributed to the analysis and verification of the clinical data. M A, A Y, J Y, J M, and S T contributed to the genetic data. J Y performed English proofreading. M A drafted the original manuscript, and all co‐authors approved the final version. The authors used ChatGPT (OpenAI, San Francisco, C A), a large language model‐based AI, to assist in improving the clarity and expression of the English language in the manuscript. The authors reviewed and edited all AI‐generated content to ensure accuracy and appropriateness. The final content is the sole responsibility of the authors.

## Conflicts of Interest

The authors declare no conflicts of interest.

## Supporting information


**Figure S1** **Pedigrees of families with *MFN2* variants identified in this study**
(A–I) Pedigrees of families harboring rare *MFN2* variants. Filled symbols represent affected individuals, open symbols represent unaffected individuals, and slashed symbols indicate deceased individuals.
The genotypes are indicated below each individual when available:
“+/−” denotes heterozygous for the indicated variant, “−/−” denotes wild‐type.

**Table S1** List of *MFN2* variants of uncertain significance (VUS) detected in the current cohort
REVEL scores ≥ 0.773 were considered moderate evidence of pathogenicity, and scores between 0.644 and 0.773 were considered supporting, based on thresholds established by the ClinGen Sequence Variant Interpretation Working Group. REVEL scores below 0.644 were interpreted as not supportive. CADD scores ≥ 25.3 and BayesDel scores ≥ 0.13 were considered indicative of damaging effects. *; Denotes variants that were observed in two affected and not observed in one unaffected individual from the same family.

## Data Availability

The data that support the findings of this study are available from the corresponding author upon reasonable request.
