# Supplementary material for: Nationwide Characterization of MFN2 ‐Related CMT in 176 Japanese Patients: Clinical and Genetic Insights
Source: Ann Clin Transl Neurol. 2025 Sep 30;13(1):170–9. doi: 10.1002/acn3.70218 (PMC12790173; doi:10.1002/acn3.70218)
Supplement: Supplementary file 1 — Figure S1 Pedigrees of families with MFN2 variants identified in this study (A–I) Pedigrees of families harboring rare MFN2 variants. Filled symbols represent affected individuals, open symbols represent unaffected individuals, and slashed symbols indicate deceased individuals. The genotypes are indicated below each individual when available: “+/−” denotes heterozygous for the indicated variant, “−/−” denotes wild‐type. [file ACN3-13-170-s001.pdf]

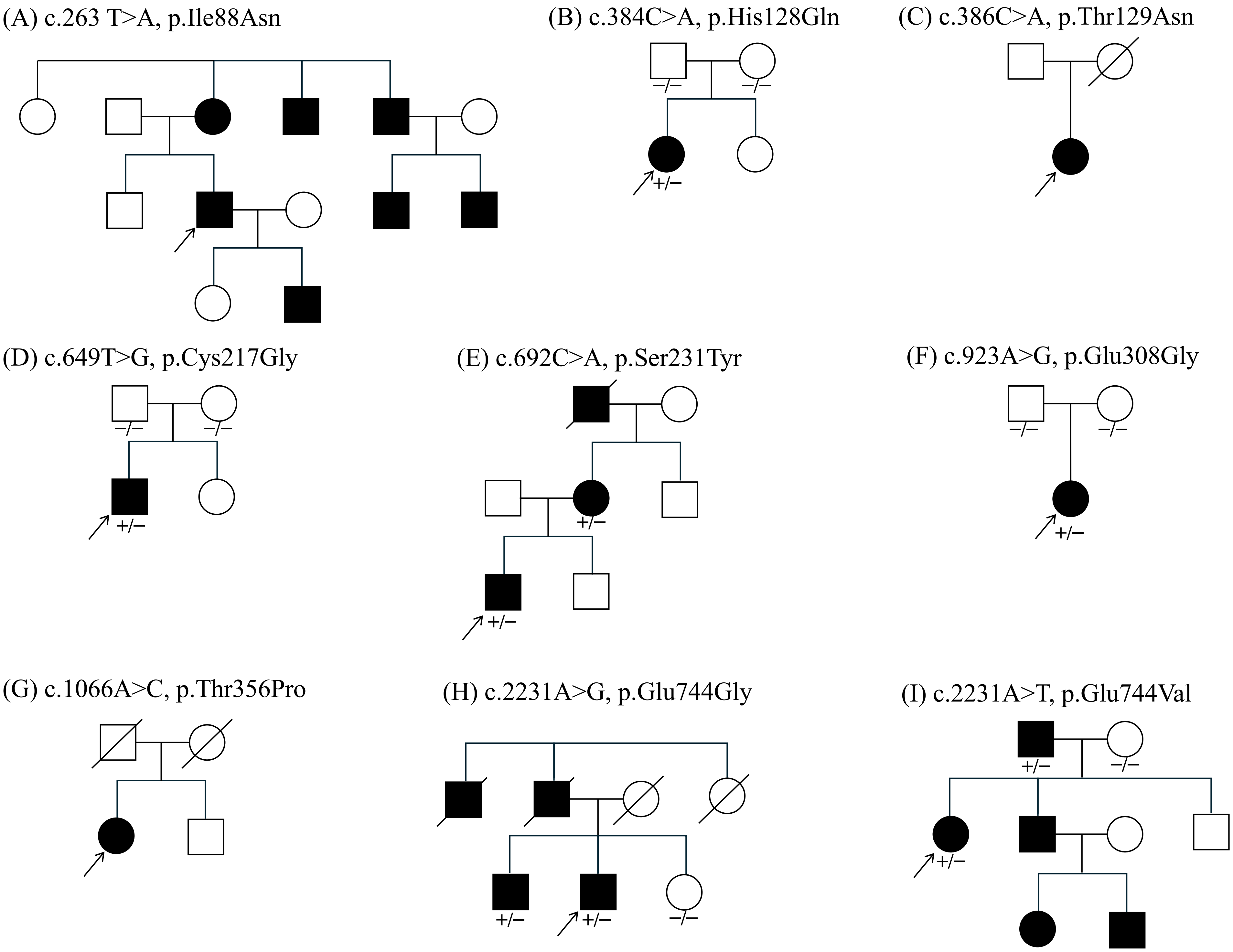

(A) c.263 T>A, p.Ile88Asn
(B) c.384C>A, p.His128Gln
−/−
−/−
+/−
(C) c.386C>A, p.Thr129Asn
(D) c.649T>G, p.Cys217Gly
−/−
−/−
+/−
(E) c.692C>A, p.Ser231Tyr
+/−
+/−
(F) c.923A>G, p.Glu308Gly
−/−
−/−
+/−
(G) c.1066A>C, p.Thr356Pro
(H) c.2231A>G, p.Glu744Gly
+/−
+/−
−/−
(I) c.2231A>T, p.Glu744Val
+/−
−/−
+/−
